# The effect of eight different gene polymorphisms on osteopenia and osteoporosis in the Turkish population

**DOI:** 10.1590/1806-9282.20241624

**Published:** 2025-05-02

**Authors:** Naim Uzun, Ahmet Kiziltunc, Adem Keskin

**Affiliations:** 1 Agri Ibrahim Cecen University, Faculty of Pharmacy, Department of Clinical Pharmacy, Department of Pharmacy Vocational Sciences – Ağrı, Turkey.; 2 Ataturk University, Faculty of Medicine, Department of Medical Biochemistry, Department of Basic Medical Sciences – Erzurum, Turkey.; 3 Aydın Adnan Menderes University, Institute of Health Sciences, Department of Biochemistry (Medicine) – Aydın, Turkey.; 4 Aydın Adnan Menderes University, Aydın Vocational School of Health Services, Department of Medical Services and Techniques – Aydın, Turkey.

**Keywords:** Gene polymorphism, Osteoprotegerin, Osteoporosis, Vitamin D receptor, Osteopenia

## Abstract

**OBJECTIVE:**

Bone mineral density is affected by many gene regions. Osteoporosis is a disease that occurs due to decreased bone mineral density and has a polygenetic multifactorial pathogenesis. The aim of this study was to examine the effect of gene variants in eight gene regions related to bone mineral density in patients diagnosed with osteopenia or osteoporosis.

**METHODS:**

A total of 60 patients diagnosed with osteoporosis, 50 patients diagnosed with osteopenia, and 40 healthy volunteers (control group) were included in the study. Collagen type I alpha 1 1997G/T, estrogen receptor α PvuII, estrogen receptor α XbaI, vitamin D receptor BsmI, lactase gene, osteoprotegerin G209A, osteoprotegerin T245G, and interleukin-6 G174C gene variants were analyzed.

**RESULTS:**

No important difference was found in the distribution of collagen type I alpha 1 1997G/T, estrogen receptor α PvuII, estrogen receptor α XbaI, vitamin D receptor BsmI, lactase gene T13910C, osteoprotegerin T245G, and interleukin-6 G174C gene variants between groups. A significant difference was detected between the distribution of osteoprotegerin G209A gene variants in the patient groups and the distribution of osteoprotegerin G209A gene variants in the control group. On the other hand, no important difference was detected in the distribution of osteoprotegerin G209A gene variants between patient groups.

**CONCLUSION:**

The osteoprotegerin G209A gene variant may be associated with the risk of osteopenia and osteoporosis in the Turkish population. Other gene variants analyzed that affect bone mineral density were not associated with the risk of osteopenia and osteoporosis.

## INTRODUCTION

Osteoporosis, characterized by skeletal fragility associated with reduced bone mass, is a well-identified and expanding public health issue. According to a report by a foundation based on osteoporosis disease, it is estimated that there are 10.2 million people with osteoporosis in America and an additional 43.4 million people have low bone mass. It is also predicted that the number of individuals with low bone mass or osteoporosis will exceed 71 million by 2030^1^. Osteoporosis, in which fractures cause significant morbidity and mortality, occurs most frequently in postmenopausal women, but cases of osteoporosis also occur in premenopausal women and men. While the treatment of cases of osteoporosis often focuses on the underlying condition, a variety of secondary causes, from endocrine diseases to genetic conditions, are often neglected^2^.

Many genes affect bone mineral density, a polygenetic skeletal trait^3^. Osteoporosis, which occurs due to decreased bone mineral density, has a polygenetic and multifactorial pathogenesis, and therefore it cannot be adequately diagnosed clinically and hence inadequately treated^4^. Therefore, it is very important to identify people at a high risk of osteoporotic fractures in a timely manner. Accordingly, screening for polygenic risk scores and similar risk factors is one of the developments in case finding^5^.

Collagen type I alpha 1 (COL1A1) is one of the two genes encoding collagen type I. Collagen type I, the most plentiful protein in people, also contributes 90% of the entire organic portion of the matrix of bones^6^. Estrogen receptor α (ESR1) stimulates the gene transcription of sialoprotein, an acidic glycoprotein specific to bone mineralized tissues that is expressed in the early stages of bone tissue development^7^. Vitamin D receptor (VDR) gene variants are known as the main risk factor for low bone mineral density^8^.

Osteoprotegerin (OPG) is a soluble nuclear factor-kappa B ligand (RANKL) decoy receptor produced predominantly by osteoblasts. It prevents osteoclastic bone resorption and osteoclast formation by inhibiting the RANKL–RANKL receptor interaction. The mass loss of bone tissues depends on the RANK–RANKL–OPG system, which is the main regulatory system of the survival, activation, and induction of osteoclast differentiation^9^. Lactase gene (LCT) variants may affect calcium intake and therefore bone health^10^. Interleukin 6 (IL-6) promotes osteoclast formation and bone resorption. Polymorphisms in the IL-6 promoter region have been associated with plasma IL-6 levels. Furthermore, variation at the IL-6 locus may contribute to genetic susceptibility to bone fragility.^11^.

Many genetic factors affect bone mineral density in different ways. This study was planned to investigate the relationship between variants observed in eight different gene regions and the pathogenesis of osteoporosis and osteopenia.

## METHODS

### Study design

Ethics committee approval for this study was received from the Non-Interventional Clinical Research Ethics Committee, Faculty of Medicine, Ataturk University (Ethics Committee approval number: 26.04.07/01/07). Sixty patients diagnosed with osteoporosis and fifty patients diagnosed with osteopenia at the Department of Physical Therapy and Rehabilitation, Faculty of Medicine Research Hospital, Ataturk University, were included in the study. In addition, 40 healthy volunteers with normal bone mineral density were included in the study. Approximately 10 mL of blood sample was taken from the participants’ left brachial vein.

### Biochemical processes

DNAs were isolated from the whole blood samples taken from all participants in the Molecular Analysis Laboratory of the Department of Medical Biochemistry, Faculty of Medicine, Atatürk University. The isolated DNAs were amplified by the polymerase chain reaction multiplex method using specific primers. Microchip hybridization using oligonucleotide probes was applied to amplicons. COL1A1 1997G/T, ESR1 PvuII, ESR1 XbaI, VDR BsmI, LCT T13910C, IL-6 G174C, OPG G209A, and OPG T245G gene regions were analyzed for variants.

### Statistical analysis

The Statistical Package for the Social Sciences for Windows 22.0 (IBM, NY, USA) program was used in the statistical analysis of evaluating the data in the study. Categorical data were expressed as percentage and frequency. Continuous data were expressed as mean±standard deviation. In group comparisons, the chi-square test was used for categorical data and the independent samples t-test was used for continuous data.

## RESULTS

The mean age of the osteoporosis patient group included in the study was 60.12±9.74 years, the mean age of the osteopenia patient group was 51.98±11.63 years, and the mean age of the control group was 43.50±12.09 years. Notably 93.33% (n=56) of the osteoporosis patient group, 90.00% (n=45) of the osteopenia patient group, and 77.50% (n=31) of the control group were women. There was a statistically significant difference in mean ages between the groups (p<0.001). Additionally, the ratio of female individuals in the osteoporosis patient group was higher than the ratio of female individuals in the control group (p=0.021). The genotype distribution of the groups is presented in Table 1.

**Table 1:** The genotype distribution of the groups.

Genotype		Osteoporosis patient group (1) n=60, n (%)	Osteopenia patient group (2) n=50, n (%)	Control group (3) n=40, n (%)	p		
					(1–2)	(1–3)	(2–3)
Collagen type I alpha 1 (COL1A1) 1997G/T							
	GG	40 (66.67)	30 (60.00)	25 (62.50)	>0.05	>0.05	>0.05
	GT	14 (23.33)	13 (26.00)	9 (22.50)			
	TT	6 (10.00)	7 (14.00)	6 (15.00)			
Estrogen receptor α (ESR1) PvuII							
	TT	12 (20.00)	16 (32.00)	13 (32.50)	>0.05	>0.05	>0.05
	TC	31 (51.67)	20 (40.00)	19 (47.50)			
	CC	17 (28.33)	14 (28.00)	8 (20.00)			
Estrogen receptor α (ESR1) XbaI							
	AA	22 (36.67)	23 (46.00)	22 (55.00)	>0.05	>0.05	>0.05
	AG	25 (41.67)	17 (34.00)	11 (27.50)			
	GG	13 (21.67)	10 (20.00)	7 (17.50)			
Vitamin D receptor (VDR) BsmI							
	BB	9 (15.00)	8 (16.00)	4 (10.00)	>0.05	>0.05	>0.05
	Bb	29 (48.33)	25 (50.00)	24 (60.00)			
	Bb	22 (36.67)	17 (34.00)	12 (30.00)			
Lactase gene (LCT)							
	TT	1 (01.67)	2 (04.00)	0 (00.00)	>0.05	>0.05	>0.05
	TC	2 (03.33)	0 (00.00)	1 (02.50)			
	CC	57 (95.00)	48 (96.00)	39 (97.50)			
Osteoprotegerin (OPG) G209A							
	GG	54 (90.00)	45 (90.00)	40 (100.00)	>0.05	**0.042** *	**0.048** *
	GA	6 (10.00)	5 (10.00)	0 (00.00)			
	AA	0 (00.00)	0 (00.00)	0 (00.00)			
Osteoprotegerin (OPG) T245G							
	TT	54 (90.00)	46 (92.00)	39 (97.50)	>0.05	>0.05	>0.05
	TG	6 (10.00)	4 (08.00)	1 (02,50)			
	GG	0 (00.00)	0 (00.00)	0 (00.00)			
Interleukin-6 (IL-6) G174C							
	GG	33 (55.00)	26 (52.00)	20 (50.00)	>0.05	>0.05	>0.05
	GC	21 (35.00)	22 (44.00)	19 (47.50)			
	CC	6 (10.00)	2 (04.00)	1 (02.50)			

COL1A1: collagen type I alpha 1; ESR1: estrogen receptor α; VDR: vitamin D receptor; LCT: lactase gene; OPG: osteoprotegerin; IL-6: interleukin-6.

* Statistically significant values (p<0.05) are denoted in bold.

No significant difference was found between the groups in terms of the genotype distributions of COL1A1 1997G/T, ESR1 PvuII, ESR1 XbaI, VDR BsmI, LCT T13910C, OPG T245G, and IL-6 G174C. In terms of the OPG G209A genotype distribution, no individual with an AA genotype was found in all three groups. Additionally, a significant difference was detected between the OPG G209A genotype distribution of both osteoporosis and osteopenia patient groups and the OPG G209A genotype distribution of the control group (Table 1).

## DISCUSSION

Osteoporosis, which occurs as a result of decreased bone mineral density and bone microarchitecture deterioration, is a chronic skeletal disease common all over the world. The most common form is the form that occurs due to aging and causes serious mortality and morbidity. Rare forms are monogenic; they usually begin in childhood or young adulthood. The most common of these monogenic forms is related to mutations in COL1A2 and COL1A1, two genes encoding type I collagen^12^. A systematic review on osteoporosis in postmenopausal women reported no significant relation between the COL1A1 gene variant and osteoporosis risk in Asian or Caucasian women^13^. Another systematic review based on the clinical studies of osteoporosis reported that women with the COL1A1 1997G/T GG genotype had higher hip bone mineral density than women with the GT genotype, but COL1A1 gene variants alone are unlikely to play a role in the association with osteoporosis and fractures^14^.

In this study conducted on the Turkish adult population, no difference was found in the distribution of the COL1A1 1997G/T gene variant between osteoporosis and osteopenia patients and healthy individuals.

The association between ESR1 gene variants and postmenopausal osteoporosis has been widely studied, but findings are conflicting. In a study examining the relationship between ESR1 gene variants and postmenopausal osteoporosis in the Chinese population, it was reported that only the ESR1 rs9340799 (XbaI) variant was related to postmenopausal osteoporosis^15^. On the other side, a systematic review on 36 observational studies involving five ESR-related gene variants, including 18,487 controls and 12,507 cases, stated that there was no relation between ESR1 gene variants and osteoporosis risk^16^.

In this study, where two gene regions of the ESR1 gene were examined, no difference was determined in the distribution of ESR1 gene variants between osteoporosis and osteopenia patients and healthy individuals.

A recent systematic review indicates that VDR BsmI and TaqI gene variants may affect the risk of osteoporosis in Asians and Caucasians^17^. In another systematic review on VDR gene variants, it was stated that the VDR FokI variant in women and Indians and the VDR BsmI variant in West Asians may affect the risk of osteoporosis^18^. A study conducted on White British men associated the VDR TaqI variant with an increased risk of osteoporosis, but not the VDR BsmI and ApaI variants^19^.

In this study, which included both male and female Turkish participants, no difference was found in the distribution of the VDR BSM gene variant between osteoporotic and osteopenic patients and healthy individuals.

Intravenous administration of adipose tissue-derived stem cells reduces bone loss in mice with osteoporosis. OPG, a natural inhibitor of RANKL receptor activator, is rich in adipose tissue-derived stem cells. Therefore, OPG inhibits osteoclast differentiation and reduces gene expression related to bone resorption^20^. In a systematic review, conducted on 14 studies including 2280 healthy controls and 2383 osteoporotic patients, it was reported that the G allele of the OPG A163G variant may increase the risk of osteoporosis in Caucasians, while the C allele of the OPG G1181C variant may reduce the risk of osteoporosis in Asians. Both these effects have been reported to be observed in postmenopausal women^21^. A study on Croatian postmenopausal women reported that the OPG A163G variant may have an impact on higher bone loss^22^.

In this study, where two gene regions of the OPG gene were examined, no difference was found in the distribution of the OPG T245G gene variant between osteoporosis and osteopenia patients and healthy people. On the other hand, a difference was detected in the distribution of the OPG G209A gene variant between osteoporosis and osteopenia patients and healthy individuals.

In this study, also no difference was found in the distribution of LCT T13910C and IL-6 G174C gene variants between osteoporosis and osteopenia patients and healthy individuals.

## CONCLUSION

In this study including both male and female Turkish adult participants, COL1A1 1997G/T, ESR1 PvuII, ESR1 XbaI, VDR BsmI, LCT T13910C, OPG T245G, and IL-6 G174C gene variants were not associated with the risk of osteoporosis and osteopenia. On the other hand, the OPG G209A gene variant may be related to the risk of osteoporosis and osteopenia.
